# Lower lobe pneumonia presenting as singultus (hiccups)

**DOI:** 10.22088/cjim.9.4.403

**Published:** 2018

**Authors:** Stamatis Karakonstantis, Sofia Pitsigavdaki, Dafni Korela, Despoina Galani

**Affiliations:** 1. 2^nd^ Department of Internal Medicine, General Hospital of Heraklion “Venizeleio-Pananeio”, Leoforos Knossou, Heraklion, Greece.

**Keywords:** Hiccups, Pneumonia, Chlorpromazine, Hypotension

## Abstract

**Background:**

Persistent hiccups can be a debilitating symptom and many such patients present to the emergency department seeking relief. A variety of serious conditions have been associated with persistent hiccups. Cases of pneumonia as a cause of hiccups have been rarely described.

**Case presentation:**

A 79-year-old male patient presented to the hospital due to persistent hiccups for 4 days. Despite lack of new respiratory symptoms or fever, a chest x-ray demonstrated a left lower lobe consolidation, which was also confirmed with a chest CT. The patient was treated with levofloxacin and at 1-month follow-up hiccups had completely resolved, while a repeat chest CT demonstrated resolution of the consolidation.

**Conclusions:**

The presentation of pneumonia in elderly patients may be atypical and may lack the symptoms and signs observed in younger patients. Hiccups may be the main presenting symptom of pneumonia.

Persistent hiccups can be a debilitating symptom and individuals with persistent hiccups will often present to the emergency department for relief of their symptom. Bouts of hiccups (<48 hours) are most commonly associated with gastric distention (e.g. overeating, carbonated beverages, aerophagia, gastric distention after upper endoscopy, excessive alcohol ingestion) (1). However, persistent (48 hours to 1 month) or intractable (>1 month) hiccups may be associated with serious underlying conditions and a detailed evaluation to exclude such conditions should be undertaken (1). Although pneumonia is included in the differential diagnosis of hiccups, very few case descriptions exist (2-6). Here, we present a case of community-acquired pneumonia presenting with persistent hiccups, without any of the typical symptoms, signs or laboratory findings of pneumonia.

## Case Presentation

A 79-year-old male with a history of smoking, chronic obstructive pulmonary disease and hypertension, was transferred early in the morning to our emergency department (ED) due to syncope. A few hours earlier, the patient had visited the on-call ED for persistent hiccups that had been interfering with his sleep. A new onset atrial fibrillation was found during that visit, and the patient was given chlorpromazine for symptomatic management of hiccups and he was discharged with a prescription for rivaroxaban. At presentation to our hospital, the patient had low blood pressure (60/40 mmHg) which was attributed to chlorpromazine (7) as no other cause was found. An echocardiography did not reveal any findings that could explain the hypotension, the electrocardiogram had no findings of ischemia and serial serum troponin measurements were normal.

**Figure 1:**
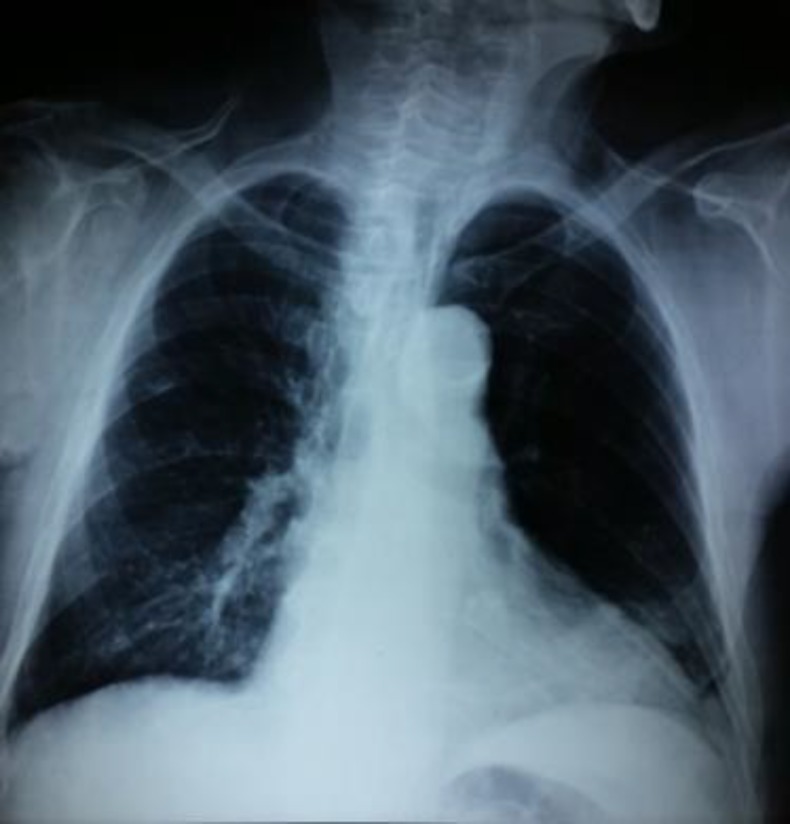
Patients Chest x-ray demonstrating a retrocardiac left lower lobe consolidation

The patient’s lab tests were normal (WBC 8200x10^9^/L, neutrophils 60%, lymphocytes 29%, eosinophil count 100/μl, normal liver function tests, sodium 136mEq/L, potassium 4.3 mEq/L, glucose 87 mg/dl, normal arterial blood gases: pO2 92 mmHg, pH 7.404, pCO2 33.5 mmHg, HCO3 20.5 mmol/L) except a slightly elevated C-reactive protein (CRP=3 mg/dL, upper limit of normal=0.5 mg/dl) and acute kidney injury (creatinine 1.61 mg/dl from a baseline of 1.1 mg/dl) which was attributed to the patient’s hypotension. The chest x-ray revealed a retrocardiac consolidation (figure 1). 

The patient had no fever. When asked, he reported cough, but not more than usual. During his hospital stay, low-grade fever was recorded (axillary temperature up to 37.3^o^C) and CRP rose up to 8.38 mg/dl. 

Given the patient’s smoking history, a brain and chest CT were performed. 

**Figure 2:**
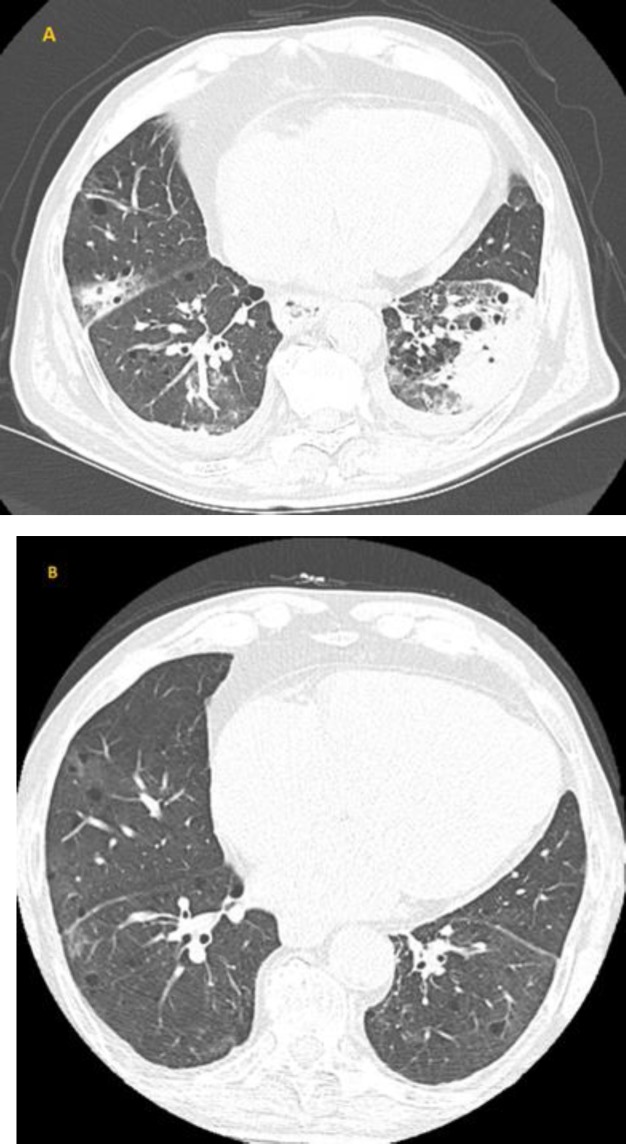
(A) The first chest CT confirmed the presence of a left lower lobe consolidation and revealed other smaller areas of consolidation in the right upper and middle lobes. (B) A follow-up CT scan in 1 month demonstrates almost a complete clearance of the previous findings

The brain CT was normal, but the chest CT revealed a left lower lobe consolidation and smaller consolidations in the right upper and middle lobes (figure 2A). The patient was prescribed levofloxacin for 5 days and baclofen as a temporary symptomatic treatment for hiccups as several physical maneuvers were unsuccessful. 

At follow-up, one month later, a second chest CT scan showed almost complete resolution of previous findings (figure 2B). The patient reported that hiccups had persisted for about a week and then gradually resolved.

## Discussion

All patients presenting to the emergency department (ED) with persistent hiccups should have at least a minimum thorough history taken, a careful physical examination, a basic laboratory screening (complete blood count, electrolytes, liver and renal studies) and a chest x-ray (1). Other investigations in the ED should be guided by findings from the history, physical examination, laboratory results and the chest x-ray. The purpose of such investigations performed in the ED should be to not miss important conditions that require prompt management. A long list of diseases can cause persistent hiccups by stimulating the components of the hiccup reflex arc (including the vagus nerve, the phrenic nerve, the thoracic part of the sympathetic chain, the C3-C5 segment of the spinal cord, and central connections among brainstem and midbrain areas) (1).

Pneumonia is a known cause of persistent hiccups, although only few case descriptions exist (2-6). Our case is interesting because the patient had no other symptoms, signs or laboratory findings typical of pneumonia and the diagnosis could have easily been missed. A previous case published in Lancet was very similar to our case. An elderly male patient presented to the hospital with persistent hiccups (of 4 days duration). He was discharged with a prescription for clonazepam. The patient was then brought back to the emergency department due to sedation presumable as a result of administration of clonazepam. A chest x-ray revealed pneumonia of the right lower lobe despite the absence of other findings suggesting pneumonia (except a slightly increased respiratory rate and a c-reactive protein of 14 mg/dl) and the patient was successfully managed with antibiotics. Of interest is that in 4 of the cases (including ours), a lower lung lobe was affected (3-6), suggesting direct irritation of the diaphragm as a potential mechanism resulting in hiccups. We did not have access to the full-text of the other case (2).

The diagnosis of pneumonia in our case was based on the radiographic evidence of pulmonary consolidation, slightly raised CRP, low-grade fever, resolution of symptoms and imaging findings with antibiotic therapy, and lack of a more likely alternative diagnosis. As highlighted by the authors of a similar case (3), pneumonia in elderly patients may lack the typical symptoms and signs of pneumonia as observed in younger patients (8). Although CRP in our case was not very high, this level of CRP elevation is common in elderly patients with pneumonia (e.g. median CRP 7.99 mg/dl in a study of elderly patients with community-acquired pneumonia (9). Another teaching point from our case is that the patients given chlorpromazine for the management of hiccups should be monitored for hypotension before being discharged from the hospital. In a previous study, significant hypotension occurred in 7 of 300 patients that received chlorpromazine (7). The authors of that study suggested starting chlorpromazine treatment with a low initial dose and monitoring the blood pressure for at least 1 hour after the first administration of the drug and after every dose increment.

In conclusion, pneumonia should be considered in patients presenting with persistent hiccups, especially in elderly patients, who may lack other symptoms and signs typical of pneumonia. Involvement of the lower lung lobes may be more likely to result in hiccups due to direct irritation of the diaphragm.
